# Supplementary material for: Drug repurposing for aging research using model organisms
Source: Aging Cell. 2017 Jun 16;16(5):1006–15. doi: 10.1111/acel.12626 (PMC5595691; doi:10.1111/acel.12626)
Supplement: Supplementary file 7 — Data S1 Zip‐Archive of all report cards. [file ACEL-16-1006-s007.zip › RC_ELI.pdf]

ELI

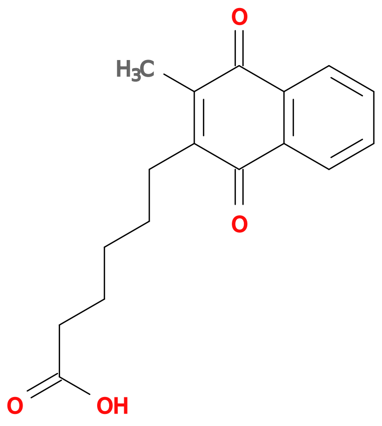

Database identifiers

ChEMBLCompound	CHEMBL135287
DrugBank	DB07714

Ranking

	Rank	Score
Drosophila	294/697	0.568
C. elegans	212/591	0.241

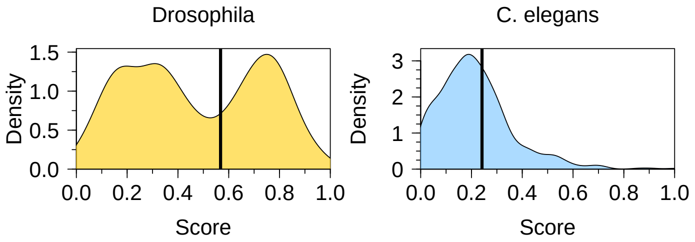

	Ageing implication	Domain conservation	Binding site conservation	Binding affinity	Bioavailability	Lipinski	Promiscuity	Purchasability	Drug approval	**Total**
Drosophila	1.0	0.801	0.861	0.795	(0.9)	0.0	-0.0	0.0	0.075	0.568
C. elegans	1.0	0.94	0.996	0.795	0.224	0.0	-0.0	0.0	0.075	0.241

Names

- 6-(3-METHYL-1,4-DIOXO-1,4-DIHYDRONAPHTHALEN-2-YL)HEXANOIC ACID
- 6-1,4-dihydroxy-3-(mercaptomethyl)-2-naphthylhexanoic acid
- 6-1,4-dihydroxy-3-(sulfanylmethyl)-2-naphthylhexanoic acid
- 6-1,4-dihydroxy-3-(sulfanylmethyl)naphthalen-2-ylhexanoic acid

Roles

ChEBI entry None has no roles

Status

Approved drug (according to ChEMBL)	No
Classification (according to DrugBank)	experimental
Number of Rule of 5 violations	0
Binding affinity to original target in log units (RF-Score prediction)	6.35
Burns *C. elegans* bioavailability prediction	-3.78

Compound Target Characteristics

Glutathione reductase, mitochondrial

Best gene implication in ageing for this target family came from gene P91938 annotated in UniProt release 2014_02. Annotation GO 8340 (determination of adult lifespan) was Inferred from Mutant Phenotype

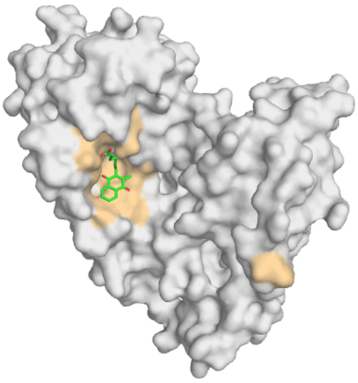

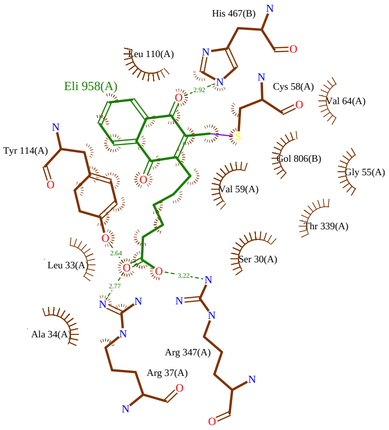

protein	amino acids contacts (binding site)												
PDB:2gh5:chainA:P00390	S	L	A	R	G	C	V	V	L	Y	T	R	H
sp:P00390:GSHR_HUMAN	S	L	A	R	G	C	V	V	L	Y	T	R	H
tr:F1LRE1:F1LRE1_RAT	-	-	-	-	-	-	-	-	L	Y	T	R	H
sp:P47791:GSHR_MOUSE	S	L	A	R	G	C	V	V	L	Y	T	R	H
sp:P91938:TRXR1_DROME	S	L	A	K	G	C	V	I	V	T	T	R	H
tr:Q86DB5:Q86DB5_CAEEL	S	I	A	R	G	C	V	V	L	Y	T	R	H
tr:Q93379:Q93379_CAEEL	S	I	A	R	G	C	V	V	L	Y	T	R	H
sp:P41921:GSHR_YEAST	S	V	A	R	G	C	V	V	L	Y	T	R	H

	whole protein		domain-based		contact-based	
protein	ident	simil	ident	simil	ident	simil
PDB:2gh5:chainA:P00390	1.0	1.0	1.0	1.0	1.0	1.0
sp:P00390:GSHR_HUMAN	1.0	1.0	1.0	1.0	1.0	1.0
tr:F1LRE1:F1LRE1_RAT	0.68	0.76	0.73	0.81	0.38	0.0
sp:P47791:GSHR_MOUSE	0.8	0.89	0.89	0.97	1.0	1.0
sp:P91938:TRXR1_DROME	0.28	0.64	0.34	0.74	0.69	0.86
tr:Q86DB5:Q86DB5_CAEEL	0.51	0.74	0.63	0.88	0.92	1.0
tr:Q93379:Q93379_CAEEL	0.5	0.73	0.63	0.88	0.92	1.0
sp:P41921:GSHR_YEAST	0.44	0.73	0.53	0.81	0.92	0.98

Trxr-1 (FBgn0020653) associated phenotypes

aging defective, developmental rate defective, lethal - all die before end of P-stage, lethal - all die before end of pupal stage, neuroanatomy defective, partially, partially lethal - majority die, some die during pharate adult stage, some die during pupal stage, stress response defective

(Information from FlyBase)

Trxr-1 (UniProt:P91938) annotation

Function: Thioredoxin system is a major player in glutathione metabolism, due to the demonstrated absence of a glutathione reductase. Functionally interacts with the Sod/Cat reactive oxidation species (ROS) defense system and thereby has a role in preadult development and life span. Lack of a glutathione reductase suggests antioxidant defense in Drosophila, and probably in related insects, differs fundamentally from that in other organisms. (PubMed:11158675, PubMed:11525742, PubMed:11796729).

Cofactor: FADNote=Binds 1 FAD per subunit.;

Biophysicochemical properties: Kinetic parameters: KM=6.5 uM for NADPH (at pH 7.4) (PubMed:11158675, PubMed:11782468, PubMed:11796729, PubMed:11877442, PubMed:17385893, PubMed:18211101, PubMed:18991392); KM=1 uM for NADPH (at pH 7.4, 2 mM EDTA, 100 mM KPO(4)) (PubMed:11158675, PubMed:11782468, PubMed:11796729, PubMed:11877442, PubMed:17385893, PubMed:18211101, PubMed:18991392); KM=1 uM for NADPH (isoform B at pH 7.4, 2 mM EDTA, 100 mM KPO(4)) (PubMed:11158675, PubMed:11782468, PubMed:11796729, PubMed:11877442, PubMed:17385893, PubMed:18211101, PubMed:18991392); KM=7.0 uM for dhd (at pH 7.4, 200 uM NADPH, 100 mM KPO(4)) (PubMed:11158675, PubMed:11782468, PubMed:11796729, PubMed:11877442, PubMed:17385893, PubMed:18211101, PubMed:18991392); KM=141 uM for dhd (at pH 7.0, 0.15 mM NADPH, 1 mM EDTA, 1 mg/ml insulin, 50 mM KPO(4), 25 degrees Celsius) (PubMed:11158675, PubMed:11782468, PubMed:11796729, PubMed:11877442, PubMed:17385893, PubMed:18211101, PubMed:18991392); KM=7 uM for dhd (at pH 7.4, 2 mM EDTA, 100 mM KPO(4)) (PubMed:11158675, PubMed:11782468, PubMed:11796729, PubMed:11877442, PubMed:17385893, PubMed:18211101, PubMed:18991392); KM=19 uM for dhd (isoform B at pH 7.4, 2 mM EDTA, 100 mM KPO(4)) (PubMed:11158675, PubMed:11782468, PubMed:11796729, PubMed:11877442, PubMed:17385893, PubMed:18211101, PubMed:18991392); KM=5.9 uM for Trx-2 (at pH 7.4, 100 uM NADPH, 2 mM EDTA, 100 mM KPO(4)) (PubMed:11158675, PubMed:11782468, PubMed:11796729, PubMed:11877442, PubMed:17385893, PubMed:18211101, PubMed:18991392); KM=310 uM for 5,5'-dithiobis-2-nitrobenzoic acid (DTNB) (at pH 7.4, 100 uM NADPH, 100 mM KPO(4)) (PubMed:11158675, PubMed:11782468, PubMed:11796729, PubMed:11877442, PubMed:17385893, PubMed:18211101, PubMed:18991392); KM=0.17 mM for DTNB (at pH 7.0, 0.2 mM NADPH, 10 mM EDTA, 100 mM KPO(4), 25 degrees Celsius) (PubMed:11158675, PubMed:11782468, PubMed:11796729, PubMed:11877442, PubMed:17385893, PubMed:18211101, PubMed:18991392); KM=380 uM for DTNB (at pH 7.4, 2 mM EDTA, 100 mM KPO(4)) (PubMed:11158675, PubMed:11782468, PubMed:11796729, PubMed:11877442, PubMed:17385893, PubMed:18211101, PubMed:18991392); KM=410 uM for DTNB (isoform B at pH 7.4, 2 mM EDTA, 100 mM KPO(4)) (PubMed:11158675, PubMed:11782468, PubMed:11796729, PubMed:11877442, PubMed:17385893, PubMed:18211101, PubMed:18991392); KM=675 uM for methylseleninate (100 uM NADPH) (PubMed:11158675, PubMed:11782468, PubMed:11796729, PubMed:11877442, PubMed:17385893, PubMed:18211101, PubMed:18991392); Vmax=24.3 umol/min/mg enzyme toward NADPH (at pH 7.4) (PubMed:11158675, PubMed:11782468, PubMed:11796729, PubMed:11877442, PubMed:17385893, PubMed:18211101, PubMed:18991392); Vmax=16 umol/min/mg enzyme toward Trx-2 (at pH 7.4, 100 uM NADPH, 2 mM EDTA, 100 mM KPO(4), 25 degrees Celsius) (PubMed:11158675, PubMed:11782468, PubMed:11796729, PubMed:11877442, PubMed:17385893, PubMed:18211101, PubMed:18991392); Note=Measurements were conducted with isoform A unless noted otherwise.;
pH dependence: Optimum pH is 7.6 for isoform A with Trx-2 and NADPH as substrates. (PubMed:11158675, PubMed:11782468, PubMed:11796729, PubMed:11877442, PubMed:17385893, PubMed:18211101, PubMed:18991392);

Subunit: Homodimer. (PubMed:17385893).

Subcellular location: Isoform B: Mitochondrion.

Subcellular location: Isoform A: Cytoplasm.

Tissue specificity: During embryogenesis, expression is seen in germ cell progenitors, developing midgut, hindgut and proventriculus. (PubMed:11525742).

Developmental stage: Expressed both maternally and zygotically during all stages of development, highest expression during adult stages. (PubMed:11525742, PubMed:11796729).

Miscellaneous: The active site is a redox-active disulfide bond.
Caution: Was originally thought to be a glutathione reductase. (PubMed:9056265).

(Information from UniProt)

gsr-1 (WBGene00008117) associated phenotypes

lethal, sterile

(Information from WormBase)
